# MCL-1 and BCL-xL-dependent resistance to the BCL-2 inhibitor ABT-199 can be overcome by preventing PI3K/AKT/mTOR activation in lymphoid malignancies

**DOI:** 10.1038/cddis.2014.525

**Published:** 2015-01-15

**Authors:** G S Choudhary, S Al-harbi, S Mazumder, B T Hill, M R Smith, J Bodo, E D Hsi, A Almasan

**Affiliations:** 1 Department of Cancer Biology, Lerner Research Institute, Cleveland Clinic, Cleveland, OH 44195, USA; 2 Department of Pathology, Case Western Reserve University School of Medicine, Cleveland, OH 44106, USA; 3 Department of Hematology and Oncology, Taussig Cancer Institute, Cleveland Clinic, Cleveland, OH 44195, USA; 4 Department of Clinical Pathology, Institute of Pathology and Laboratory Medicine, Cleveland Clinic, Cleveland, OH 44195, USA

## Abstract

Overexpression of anti-apoptotic BCL-2 family members is a hallmark of many lymphoid malignancies, including chronic lymphocytic leukemia (CLL) and non-Hodgkin lymphoma (NHL) that can be targeted with small molecule inhibitors. ABT-199 is a rationally designed BCL-2 homology (BH)-3 mimetic that specifically binds to BCL-2, but not to MCL-1 and BCL-xL. Although the thrombocytopenia that occurs with navitoclax treatment has not been a problem with ABT-199, clinical trials in CLL could benefit by lowering the ABT-199 concentration through targeting other survival pathways. In this study, we investigated the mechanisms of resistance that develops to ABT-199 therapy by generating ABT-199-resistant (ABT199-R) cell lines via chronic exposure of NHL cell lines to ABT-199. Acquired resistance resulted in substantial AKT activation and upregulation of MCL-1 and BCL-xL levels that sequestered BIM. ABT199-R cells exhibited increased MCL-1 stability and failed to activate BAX in response to ABT-199. The ABT-199 acquired and inherent resistant cells were sensitized to treatment with ABT-199 by inhibitors of the PI3K, AKT, and mTOR pathways, NVP-BEZ235 and GS-1101. NVP-BEZ235, a dual inhibitor of p-AKT and mTOR, reduced MCL-1 levels causing BIM release from MCL-1 and BCL-xL, thus leading to cell death by BAX activation. The PI3K*δ* inhibitor GS-1101 (idelalisib) downregulated MCL-1 and sensitized ABT199-R cells through AKT-mediated BAX activation. A genetic approach, through siRNA-mediated down-regulation of AKT, MCL-1, and BCL-xL, significantly decreased cell survival, demonstrating the importance of these cell survival factors for ABT-199 resistance. Our findings suggest a novel mechanism that modulates the expression and activity of pro-survival proteins to confer treatment resistance that could be exploited by a rational combination therapeutic regimen that could be effective for treating lymphoid malignancies.

Diffuse large B-cell lymphoma (DLBCL), the most common subtype of non-Hodgkin lymphoma is categorized as germinal center B-cell-like and activated B-cell-like disease.^1^ Several gene expression-profiling studies have shown distinct molecular signatures in germinal center and activated B-cell-like disease that differentiate them based on oncogenic dependency and clinical outcome of the disease.^2, 3^ A hallmark pathway that drives DLBCL tumor progression is mutation in immunoglobulin heavy variable gene rearrangement, causing activation of the B-cell receptor pathway that increases expression of specific receptors that facilitate activation of critical pathways involved in tumor progression and upregulation of anti-apoptotic BCL-2 family proteins, thereby causing chemoresistance and aggressive relapse in the clinic.^4, 5, 6, 7^

The role of constitutive PI3K signaling in B cells, particularly of the PI3K*δ* isoform that is primarily expressed in hematopoietic cells, has been implicated as a central mechanism for relaying cell survival, adhesion, and proliferative signals. PI3K*δ via* AKT achieves transcriptional, translational, and posttranslational regulation of BCL-2 family proteins by regulating mTOR, GSK3, FOXO, and NF-*κ*B.^5, 8^ The BCL-2 family members are key regulators of the intrinsic apoptotic pathway and are classified into three classes of proteins based on their structural similarity and function: the anti-apoptotic proteins (BCL-2, BCL-xL, MCL-1, BFL-1, and BCL-w) sequester the BH3-only proteins (BIM, BID, PUMA, and NOXA) that in turn activate the pro-apoptotic proteins (BAX and BAK). In some cases, anti-apoptotic BCL-2 proteins can also sequester pro-apoptotic proteins. BAX/BAK oligomerization causes mitochondrial outer membrane permeabilization, resulting in cytochrome *c* release and apoptosis.^9, 10^ Chronic lymphocytic leukemia (CLL) cells rely on increased expression of anti-apoptotic BCL-2 proteins; strategies to restore apoptosis by antagonizing them have led to development of BH3 mimetics as therapeutic agents that have a robust clinical response with reduced toxicity.^9, 11^

ABT-737 (clinical derivative, navitoclax or ABT-263) is a small-molecule inhibitor that binds to the BH3 domain of BCL-2, BCL-xL, and BCL-w, releasing BH3-only proteins and causing mitochondrial outer membrane permeabilization via BAX/BAK activation.^12, 13, 14^ Our previous studies with primary CLL samples showed that the inability of ABT-737 to cause cell death in patient-derived samples correlated with high levels of MCL-1 and BFL-1 expression.^15^ Moreover, navitoclax caused on-target toxicity in BCL-xL-dependent platelets, causing thrombocytopenia in CLL patients.^16^ This led to the re-engineering of navitoclax into a potent and orally bioavailable BCL-2-specific inhibitor, ABT-199, which demonstrates robust anti-leukemic activity toward BCL-2- but not BCL-xL-dependent tumors.^17, 18, 19, 20, 21^ Studies with primary patient samples of CLL, acute lymphoblastic leukemia, and mouse xenograft models have shown that prolonged dosing of ABT-199 not only maintains robust antagonism towards BCL-2 but also spares platelets, thus avoiding thrombocytopenia.^17, 19, 22^

Preliminary data from clinical trials with ABT-199 have shown high response rates in CLL. However, in refractory CLL, initial results of ABT-199 treatment have shown potential for tumor lysis syndrome, requiring slow dose escalation.^17, 18, 23^ Binding affinity studies with fluorescence polarization assay and TR-FRET showed that ABT-199 has very weak affinity for BCL-xL and MCL-1. Correspondingly, cell viability assays with non-Hodgkin lymphoma cell lines have shown that ABT-199 has limited efficacy in BCL-xL- and MCL-1-dependent hematopoietic malignancies.^17^

Acquired and inherent drug resistance is always a potential concern associated with even the most effective chemotherapeutic drugs, impeding their progression in clinical trials for use as single agents. Therefore, here we investigated the mechanisms of ABT-199-resistance in sensitive B-cell lymphoid cell lines after chronic exposure to ABT-199. Our results indicate that acquired ABT-199-R develops because of increased activation of the PI3K/AKT/mTOR signaling pathways and upregulation of MCL-1 and BCL-xL that sequester BIM. A combination approach using PI3K inhibitors and ABT-199 sensitized inherent and acquired ABT-199-R cells by targeting critical survival pathways upstream of MCL-1 and BCL-xL. Our data reveal novel mechanistic insights into the role of MCL-1 and BCL-xL in ABT-199-resistance and provide rational combination strategies to overcome it in lymphoid malignancies.

## Results

### DLBCL cells with low BCL-xL and MCL-1 expression develop resistance to ABT-199 following chronic exposure

**Figure 1:**
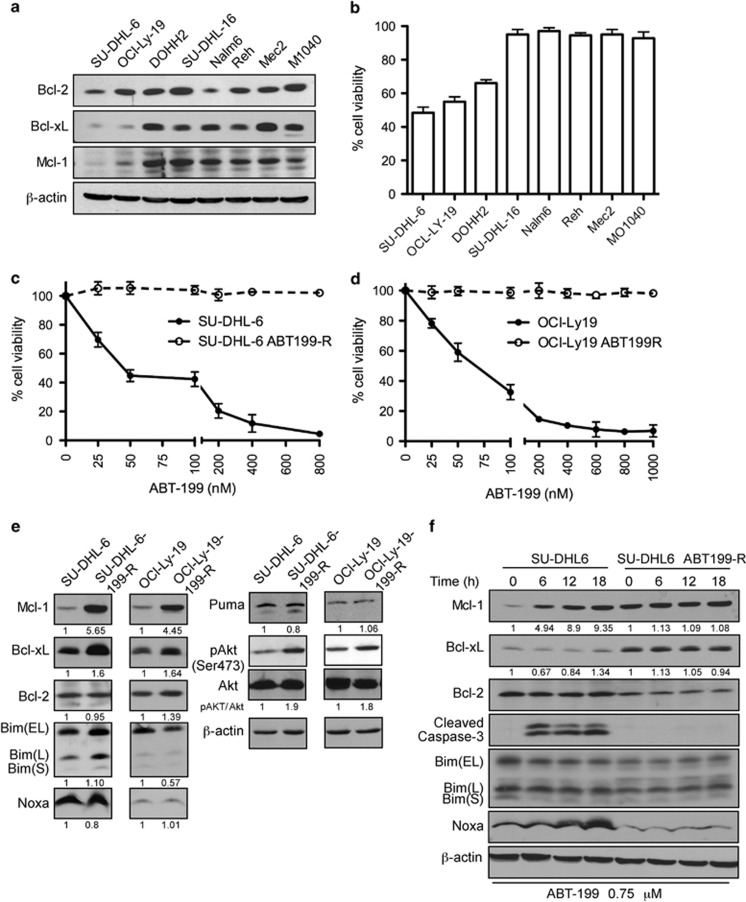
DLBCL cells with low MCL-1 and/or BCL-xL expression are sensitive to ABT-199 and develop resistance after chronic ABT-199 exposure by upregulating MCL-1, BCL-xL, and p-AKT levels. (**a**) Expression levels of the pro-survival proteins MCL-1, BCL-2, and BCL-xL in DLBCL (SU-DHL-6, OCL-LY-19, SU-DHL-16), follicular lymphoma (DOHH2), acute lymphoblastic leukemia (Nalm6, Reh), and CLL (Mec2, MO1040) cell lines. *β*-actin was used as the loading control. (**b**) The indicated cell lines were treated with 50 nM ABT-199 for 48 h. The viability shown represents the percentage of live cells relative to control cells treated with dimethyl sulfoxide. Parental and ABT-199-R (**c**) SU-DHL-6 and (**d**) OCl-Ly-19 cells were treated with the indicated concentrations of ABT-199 for 48 h and cell viability was determined by Annexin V-PI staining. Control cells were treated with dimethyl sulfoxide. (**e**) Expression levels of: (i) pro-survival proteins MCL-1, BCL-2, and BCL-xL, (ii) pro-apoptotic proteins BIM, NOXA, and PUMA, and (iii) total AKT and p-AKT (Ser473) in untreated parental and ABT199-R-derivative DLBCL cell lines at the indicated time. SU-DHL-6 parental and resistant cells. Both panels represent one experiment with BCL-2 and *β*-actin serving as loading controls. (**f**) Cells were treated with ABT-199 for the indicated time. MCL-1, BCL-2, BCL-xL, BIM, NOXA, PUMA, and cleaved caspase-3 were determined by immunoblotting. *β*-actin was used as a loading control. Standard deviation (S.D.) is indicated in **b**–**d** as error bars (*N*=3). The experiments in **a**, **e**, and **f** are representative of three independent experiments. Numbers below blots indicate increase in protein levels as determined by ImageJ quantification, which in case of pAKT was normalized to total AKT levels

ABT-199 has high affinity to bind to BCL-2 at sub-nanomolar concentrations as compared with MCL-1 and BCL-xL. Analyzing levels of BCL-2 family proteins in representative cell lines from various B-cell malignancies showed a high variability in expression, with SU-DHL-6 and OCL-LY-19 cells having low levels of MCL-1 and BCL-xL (Figures 1a and b). Subsequent cell viability analysis in these two DLBCL cell lines by Annexin V-propidium iodide (PI) assay demonstrated a high sensitivity to ABT-199, with an IC_50_ of 50 nM for SU-DHL-6 and 70 nM for OCL-LY-19 (Figures 1c and d). To determine whether these sensitive tumor cells would develop ABT-199-resistance in response to chronic ABT-199 exposure, we generated ABT-199-resistant (ABT-199-R) cell lines as described in Materials and Methods. The resistance thus developed was verified by exposing the parental cells, SU-DHL-6, OCL-LY-19, and their resistant derivatives to increasing concentrations of ABT-199 for 48 h and then analyzing cell viability. Although parental cell lines with low BCL-xL and MCL-1 expression were very sensitive to ABT-199, resistant sub-clones were resistant to ABT-199 at concentrations fourfold higher than the IC_50_ of the parental cells.

### ABT-199-R correlates with increase in p-AKT, MCL-1, and BCL-xL levels

To investigate the mechanism of ABT-199-resistance, we next examined the expression patterns of BCL-2 family proteins in parental and resistant cells. Immunoblot analyses show that the levels of MCL-1 and BCL-xL were higher in resistant (SU-DHL-6 ABT199-R and OCL-LY-19 ABT199-R) as compared with parental cells (Figure 1e). In contrast to our previous studies of ABT-737-resistance in acute lymphoblastic leukemia cell lines,^24^ we found no changes in BCL-2 level between parental and ABT199-R cells. There was also no change in the levels of the BH3-only proteins BIM, NOXA, and PUMA.

We next examined upstream signaling pathways known to control transcription and translation of MCL-1 and BCL-xL. Studies on DLBCL have shown that AKT and mTOR, separately or in combination, regulate transcription and/or translation of MCL-1.^25, 26, 27^ Exploring AKT activity in our experimental system revealed that p-AKT levels were upregulated 1.9-fold in SU-DHL-6 ABT199-R and 1.8-fold in OCL-LY-19 ABT199-R cells (Figure 1e). We then tested the effect of acute ABT-199 (0.75 *μ*M) treatment on the expression of BCL-2 family members on cell death. Immunoblot analyses indicate that MCL-1 protein level increased from baseline in parental SU-DHL-6, OCL-LY-19, and OCL-LY-19 ABT199-R cells, but not in the SU-DHL-6 ABT199-R derivative cells. In contrast, there were no robust changes in BCL-xL, BCL-2, and BIM levels. As expected, caspase-3 was proteolytically cleaved only in parental cells and cleavage occurred as early as 6 h (Figure 1f, Supplementary Figure S1a), indicating apoptosis activation. NOXA levels increased in SU-DHL-6 only at later time points, with no significant change observed in OCL-LY-19 cells (data not shown). These results indicate that ABT199-R cell express elevated levels of p-AKT, MCL-1, and BCL-xL and resist caspase-3 cleavage and thus avoid apoptosis activation. The increase in MCL-1, with no change in Bcl-xL levels, in sensitive parental cell lines in response to acute ABT-199 was not sufficient to rescue them from cell death, as indicated by cleaved caspase-3. These findings indicate the importance of BCL-xL expression in mediating ABT-199-resistance.

### Increased Mcl-1 and Bcl-xL mRNA levels and MCL-1 protein stability contribute to ABT-199-resistance

**Figure 2:**
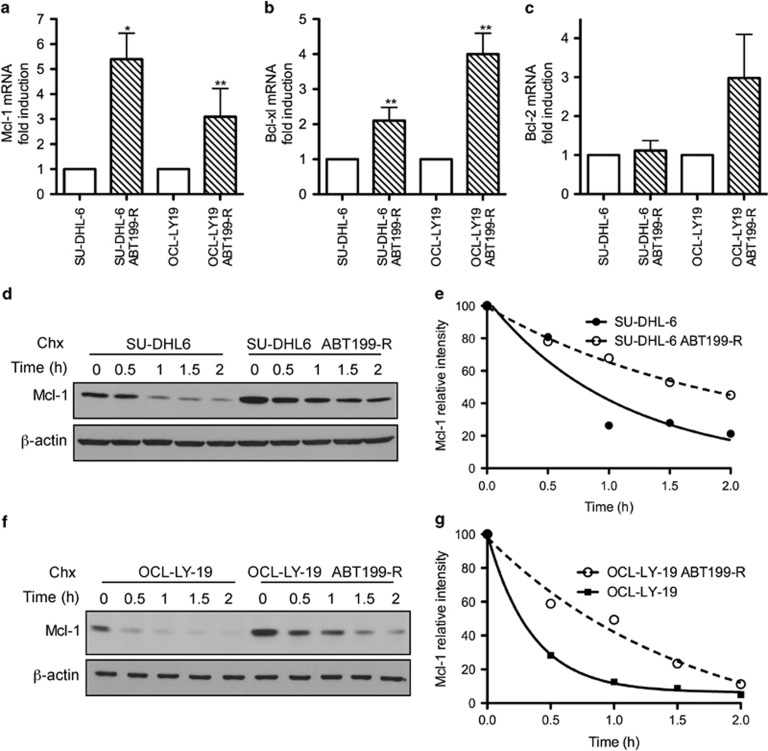
ABT-199-resistance is associated with the upregulation of Mcl-1 and Bcl-xL mRNA and increased MCL-1 protein stability. RNA was extracted from parental and ABT199-R SU-DHL-6 and OCl-LY-19 cells after culture in the absence of ABT-199 for 72 h. (**a**) Mcl-1, (**b**) Bcl-xL, and (**c**) Bcl-2 fold change was analyzed by qRT-PCR. RNA levels of parental cells were set to 1 for analysis (*P<0.04, **P<0.05). MCL-1 protein half-life was determined by treating parental and ABT199-R (**d**) SU-DHL-6 and (**f**) OCl-LY-19 cells with cycloheximide (10 *μ*g/ml) for the indicated time, followed by immunoblotting. *β*-actin was used as the loading control. Data in **e** and **g** were quantified by ImageJ. The experiments from **a** to **g** are representative of three independent experiments

We next investigated the underlying cause of the increase in MCL-1 and BCL-xL protein levels in ABT199-R cells. qRT-PCR analyses indicated that *Mcl-1* mRNA levels were increased fivefold in SU-DHL-6 ABT199-R and threefold in OCL-LY-19 ABT199-R compared with the respective parental cells. *Bcl-xL* levels were also increased by twofold in SU-DHL-6 ABT199-R and fourfold in OCL-LY-19 ABT199-R cells (Figures 2a and b). *Bcl-2* transcript levels were elevated in OCL-LY-19 ABT199-R, but not in SU-DHL-6 ABT199-R cells (Figure 2c). Following a brief incubation with ABT-199 (0.75 *μ*M), *Mcl-1*, *Bcl-xL*, and *Bcl-2* mRNA levels did not significantly change in parental or resistant SU-DHL-6 and OCL-LY-19 cells (Supplementary Figure S2).

As MCL-1 protein has a short half-life,^28^ we determined its stability by inhibiting its synthesis with cycloheximide, as before.^24^ MCL-1 half-life significantly increased in resistant cells (78 min for SU-DHL-6 ABT199-R and 69 min for OCL-LY-19 ABT199-R) as compared with parental cells (42 min for SU-DHL-6 and 15 min for OCL-LY-19) (Figures 2d and g). Taken together, these results show that elevated MCL-1 and BCL-xL levels in resistant cells could be attributed to increased mRNA levels, as well as MCL-1 protein stability, which developed in the course of acquiring ABT-199-resistance.

### Elevated MCL-1 and BCL-xL levels sequester BIM to determine ABT-199-resistance

**Figure 3:**
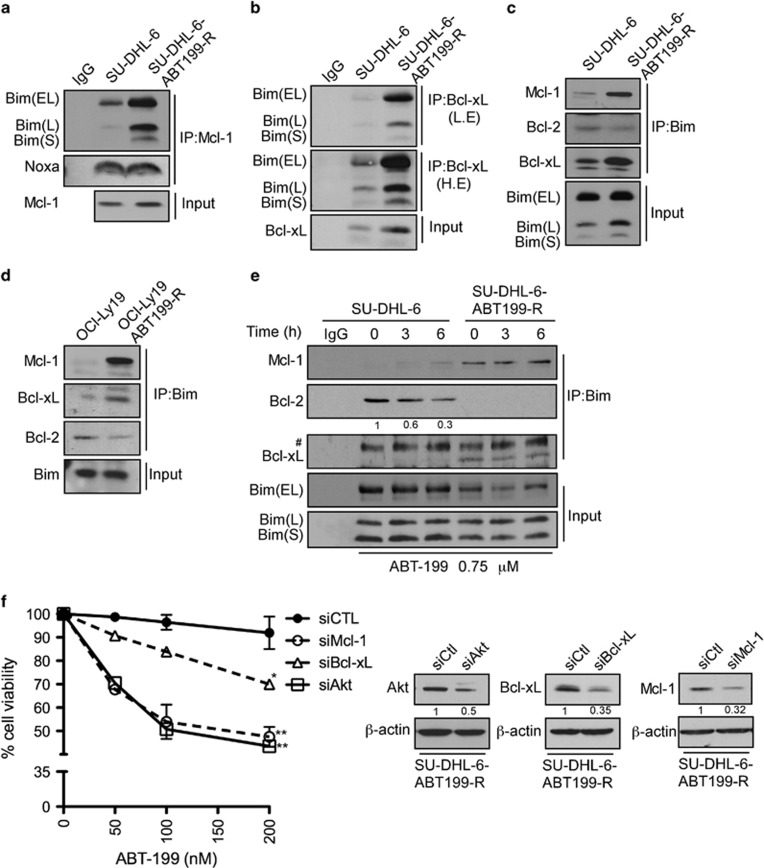
BIM associates with increased MCL-1 and BCL-xL protein in ABT199-R cells. MCL-1 and BCL-xL were immunoprecipitated with (**a**) MCL-1- and (**b**) BCL-xL-specific antibodies and their association with BIM and NOXA was probed by immunoblotting with specific antibodies. Reciprocal immunoprecipitation-western blotting for BIM was performed in (**c**) SU-DHL-6 (**d**) OCL-LY-19 parental and ABT199-R cell lines and its association with MCL-1, BCL-xL, and BCL-2 was analyzed. (**e**) Parental and resistant cells were treated with ABT-199 at the indicated time and BIM immunoprecipitates were examined by immunoblotting for association with MCL-1, BCL-xL, and BCL-2 in SU-DHL-6 parental and ABT199-R cells. (**f**) SU-DHL-6 ABT199-R cells were transfected with *siAKT*, *siMcl-1*, *siBcl-xL* or *siControl* and treated with the indicated concentration of ABT-199 for 24 h. Cell viability was determined by Annexin V-PI staining. Control cells were treated with dimethyl sulfoxide (*P<0.02, **P<0.001) (*N*=2). *β*-actin was used as loading control. The experiments from **a** to **e** are representative of three independent experiments. L.E and H.E: low and high exposure, respectively. ^#^, non-specific band

ABT-199, like its predecessor navitoclax, binds to the BH3 domain of BCL-2, displacing BIM and causing BAX activation and cell death.^17^ To understand the mechanism of acquired ABT-199-resistance, we next investigated protein–protein interactions between pro-survival MCL-1, BCL-xL, and BCL-2 proteins and the BH3-only protein BIM. MCL-1 was immunoprecipitated from parental and resistant SU-DHL-6 cells lysed with 2% CHAPS buffer. Immunoblotting analyses show increased BIM binding to MCL-1 in SU-DHL-6 ABT-99-R compared with parental cells (Figure 3a). Similarly, immunoprecipitation of BCL-xL revealed that more BIM was associated with it in ABT199-R cells (Figure 3b). Reciprocal immunoprecipitation with BIM and immunoblotting for BCL-2, MCL-1, and BCL-xL confirmed these observations. As expected, more MCL-1 and BCL-xL was associated with BIM in the resistant compared with parental cells (Figure 3c). Strikingly, less BCL-2 was bound to BIM in the resistant cells. Similar results were observed in immunoprecipitation studies performed in OCL-LY-19 parental and resistant cells (Figure 3d). Moreover, BIM immunoprecipitation from lysates of SU-DHL-6 parental and resistant cells that had undergone acute ABT-199 exposure revealed that BIM was displaced from BCL-2 by ABT-199 only in parental, but not in ABT199-R cells (Figure 3e and Supplementary Figure S1b). Taken together, these results show that elevated MCL-1 and BCL-xL levels sequester BIM that was displaced from BCL-2 during the course of acquired ABT-199-resistance. This property allowed the resistant cells to avoid the dependency on BCL-2 that is exploited by ABT-199 in parental cells. To directly demonstrate a role for MCL-1, BCL-xL, and AKT, we reduced their levels by the respective siRNAs in SU-DHL-6 ABT199-R cells (Figure 3f). Annexin V-PI analysis following ABT-199 treatment showed that two (AKT) to threefold (MCL-1 and BCL-xL) reduction in protein levels increased the sensitivity of ABT-199R cells significantly. These findings indicate that: (i) activation of the AKT pathway, and (ii) upregulation of MCL-1 and BCL-xL are key facets in mediating ABT-199-resistance.

### NVP-BEZ235, in combination with ABT-199, downregulates MCL-1 levels and sensitizes ABT199-R cells

The above data establish the important role of MCL-1 and BCL-xL in mediating ABT-199-resistance. In order to overcome acquired as well as inherent ABT199 resistance, we next selected clinically relevant pharmacological inhibitors that could target signaling pathways responsible for maintaining high MCL-1 levels. Because both p-AKT and mTOR have been reported to affect MCL-1 levels,^27, 29, 30^ we targeted these pathways with a dual PI3K/mTOR inhibitor, NVP-BEZ235, in combination with ABT-199.

**Figure 4:**
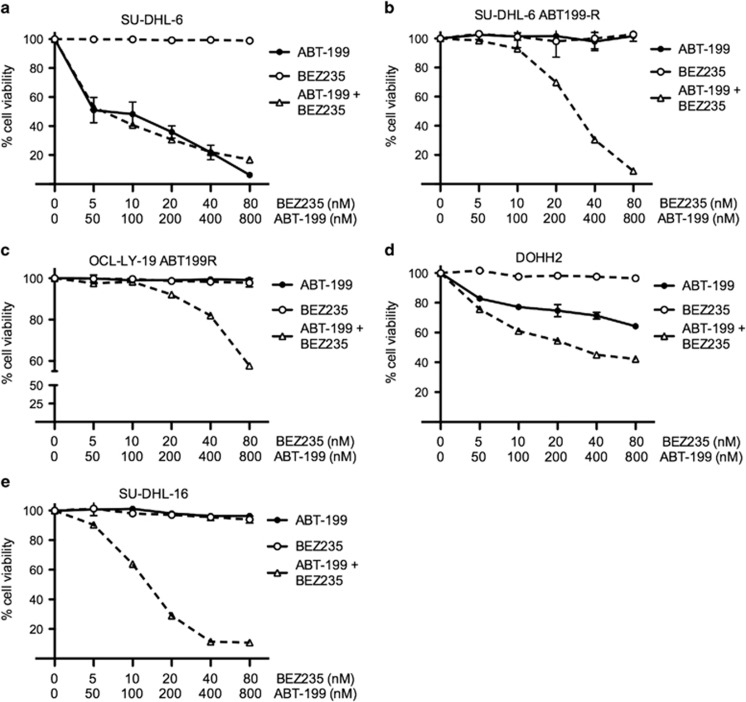
ABT-199 in combination with NVP-BEZ235 sensitizes ABT199-R cells. Parental and resistant derivative DLBCL cell lines (**a**) SU-DHL-6 (**b**) SU-DHL-6 ABT199-R (**c**) OCl-Ly-19 ABT-199R (**e**) SU-DHL-16, and (**d**) FL cell line DOHH2 were treated with the indicated concentration of ABT-199 and NVP-BEZ235, alone and in combination for 24 h. Cell viability was determined by Annexin V-PI staining represented as percentage relative to control cells treated with dimethyl sulfoxide. Standard deviation (S.D.) is indicated by the error bars (*N*=3)

**Figure 5:**
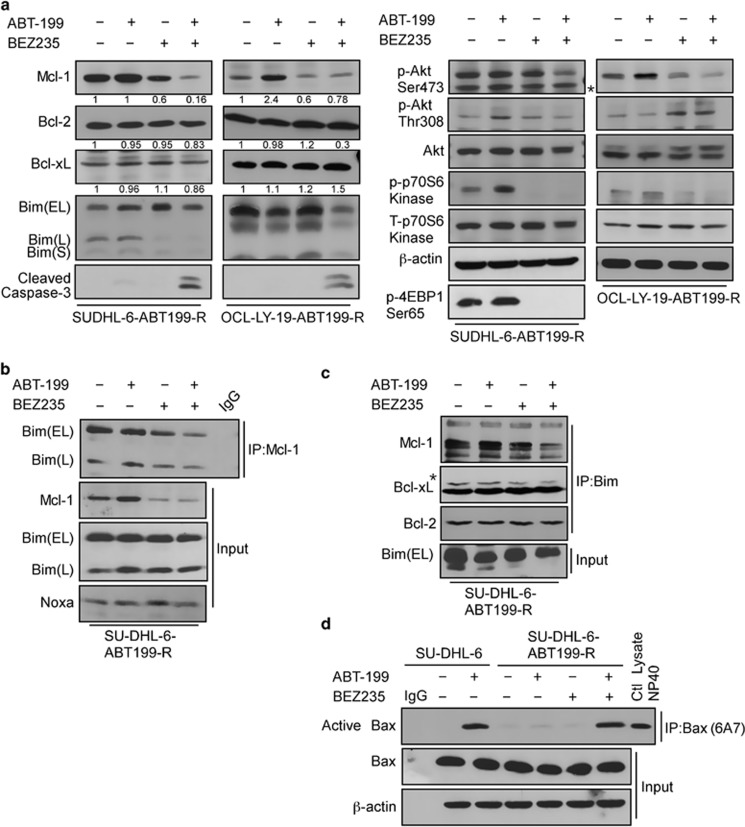
NVP-BEZ235 downregulates MCL-1 through p-AKT and mTOR inhibition and causes BAX activation following BIM release from MCL-1. (**a**) Expression of MCL-1, BCL-xL, BCL-2, BIM, p-AKT (Ser473), AKT, p-p70S6 kinase (Thr389), p-70S6 kinase, p-4EBP1 (Ser65), and cleaved caspase-3 in SU-DHL-6 ABT199-R and OCL-LY-19 ABT199-R cells treated with ABT-199 (400 nM), NVP-BEZ235 (40 nM) or in combination for 6 h. Both panels represent one experiment with BCL-2 and *β*-actin serving as loading controls. * denotes a non-specific band. SU-DHL-6 ABT199-R cells were treated with ABT-199R or NVP-BEZ235, alone or in combination, in concentrations as in a for 6 h and cells were lysed with 2% CHAPS buffer. (**b**) MCL-1 and (**c**) BIM were immunoprecipiated and their corresponding binding partners MCL-1, BIM, NOXA, BCL-2, or BCL-xL were analyzed by western blotting with specific antibodies. (**d**) Parental and resistant SU-DHL-6 cells treated with ABT-199R, NVP-BEZ235, or their combination (concentration as in a) were lysed with 1% CHAPS buffer and active BAX was immunoprecipated with BAX 6A7 and probed by BAX N20 antibodies by immunoblotting. The experiments in **a** to **d** are representative of three independent experiments

Cell viability analysis by Annexin V-PI showed high sensitivity to a 24 h incubation with the ABT199-NVP+BEZ235 combination, whether ABT-199-R was acquired (SU-DHL-6 ABT199-R and OCL-LY-19 ABT199-R) or inherent (DOHH2, SU-DHL-16). In contrast, treatment with either agent alone was not effective (Figures 4b and e). Moreover, no additional cell death occurred with this combination treatment in parental cell lines when compared with ABT-199 treatment alone (Figure 4a). As this combination induced maximum cell death at 24 h, an earlier 6 h time point was further chosen to study the mechanism of action of this combination treatment. ABT199-R cells were treated with ABT-199 (400 nM) and NVP-BEZ235 (40 nM), alone or in combination. Interestingly, in ABT199-R cells, MCL-1 expression, which had declined after NVP-BEZ235 treatment alone, further decreased with the combination treatment, despite the fact that ABT-199 alone increased MCL-1 levels (Figures 1e, f, and 5a). In contrast, BCL-2 and BCL-xL levels did not change. In accordance with the cell viability data, caspase-3 was activated only following the combination treatment, suggesting that resistant cells depended on MCL-1 for their survival.

Evaluation of signaling proteins in the PI3K/mTOR pathway showed a decrease in active AKT that was more pronounced following the ABT-199+NVP-BEZ235 combination. Because NVP-BEZ235 also targets mTOR, we next examined the activity of the downstream targets of mTORC1. Phosphorylation of p-70S6 kinase and 4-EBP1 was inhibited not only after treatment with NVP-BEZ235 alone but also when it was used in combination with ABT-199. Total AKT and p-70S6 kinase levels did not significantly change (Figure 5a). These findings indicate that a decrease in MCL-1 protein expression in response to the ABT-199+NVP-BEZ235 combination was associated with decreased activity of AKT and mTOR. Next, we investigated the mechanism of sensitization by this combination by examining the association between MCL-1 or BCL-xL and BIM. MCL-1 immunoprecipitation revealed that following the combination treatment, it was no longer associated with BIM (Figure 5b), and BIM and NOXA expression was not changed. The reciprocal immunoprecipitation with BIM indicated a decreased association of BIM with MCL-1 (Figure 5c). The BIM association with BCL-xL was decreased after NVP-BEZ235-only treatment that was further reduced with the ABT-199 and NVP-BEZ235 combination.

It has been reported in Bax^−/−^, Bak^−/−^ mouse embryonic fibroblasts^17^ that ABT-199 is ineffective, because Bax and Bak are essential for activating apoptosis.^31^ Acute ABT-199 treatment led to BAX activation in parental SU-DHL-6 but not in ABT199-R-derivative cells. These results indicate that BAX activation in response to ABT-199 is impaired in ABT-199-R cells (Figure 5d). Interestingly, BAX was activated with the ABT-199+NVP-BEZ235 combination in ABT199-R cells, but not with the individual treatments, corresponding to a decrease in MCL-1 levels (Figures 5a and d). These findings further highlight the resistant cell's dependency on MCL-1 to avoid BAX activation. Because NOXA level did not increase with the combination treatment in ABT199-R cells, we can conclude that disruption of the BIM/MCL-1 association was not due to an increase in NOXA/MCL-1 association, but was rather a result of a decrease in PI3K/mTOR activity and downregulation of MCL-1. The inhibition of the PI3K/mTOR pathway by NVP-BEZ235 alone was not sufficient to cause cell death; however, it potentiated the effect of ABT-199.

### ABT-199 in combination with GS-1101 sensitizes ABT-199-R cells by targeting the PI3K pathway

**Figure 6:**
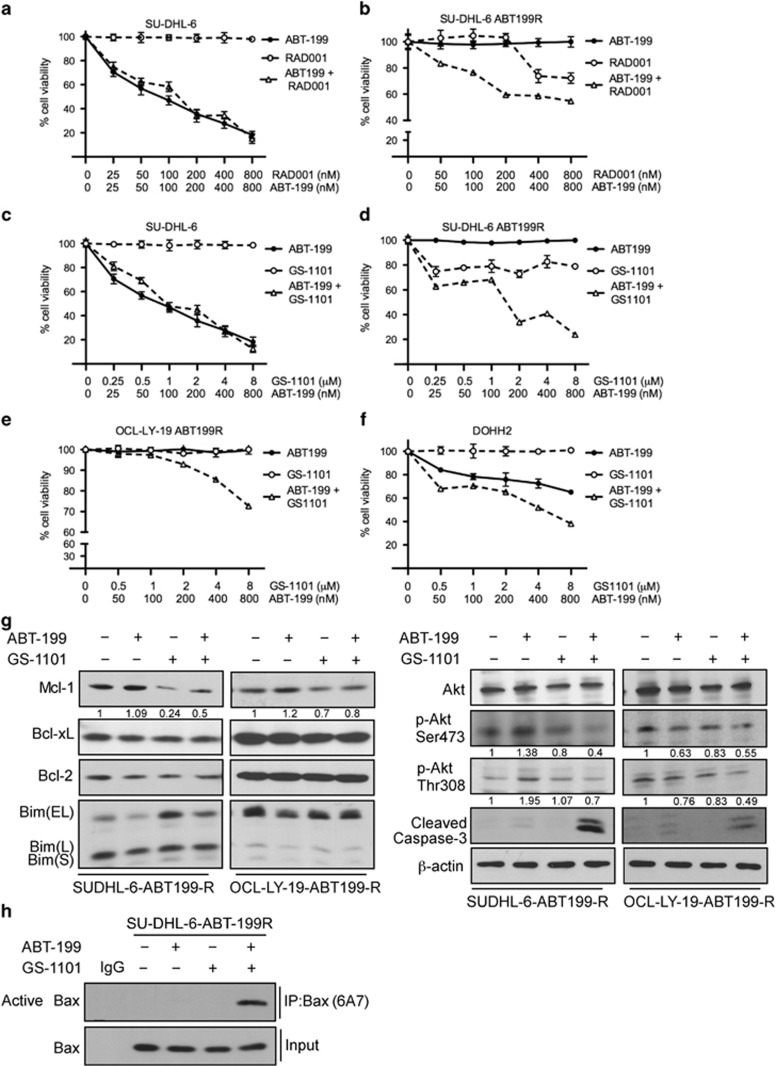
GS-1101 in combination with ABT-199 targets the PI3K pathway, sensitizing ABT-199-R cells by MCL-1 downregulation and BAX activation. Parental and ABT-199-R-derivative DLBCL cell lines SU-DHL-6 (**a**–**d**), OCL-LY-19 ABT199-R (**e**) and the FL cell line DOHH2 (**f**) were treated for 24 h with the indicated concentration of ABT-199, RAD001, and GS-1101, alone or in combination. Cell viability was determined by staining with Annexin V-PI, and represented as the percentage relative to control cells treated with dimethyl sulfoxide. Standard deviation (S.D.) is indicated in **a**–**e** by error bars (*N*=3). (**g**) Expression levels of MCL-1, BCL-xL, BCL-2, BIM, p-AKT (Ser473), p-AKT (Thr308), AKT, and cleaved caspase-3 in SU-DHL-6 ABT199-R cells treated with ABT-199 (400 nM) and GS-1101 (4 *μ*M) alone or in combination, and OCL-LY-19 ABT199-R treated with ABT-199 (400 nM) and GS-1101 (8 *μ*M), alone or in combination, for 24 h. *β*-actin was used as the loading control. (**h**) ABT-199-R SU-DHL-6 cells treated with ABT-199R (400 nM), GS-1101 (4 *μ*M), alone or in combination, were lysed with 1% CHAPS buffer, and active BAX was immunoprecipated with BAX 6A7 and detected using BAX N20 antibodies following immunoblotting. The experiments in **g** and **h** are representative of three independent experiments

Given that the dependency of ABT-199-R cells on MCL-1 is regulated by the PI3K/mTOR pathway, we next asked whether these cells could be sensitized to ABT-199 by targeting the PI3K or mTOR pathways. We used the PI3K*δ* and mTORC1 inhibitors, GS-1101 and RAD001, respectively, as before,^32, 33^ in combination with ABT-199. Cell viability analyses with Annexin V-PI indicated that GS-1101 in combination with ABT-199 sensitized SU-DHL-6 ABT199-R cells more than the RAD001+ABT-199 combination (Figures 6a and d). The latter combination was effective only at a higher concentration of RAD001 that may not be achieved in clinical settings. Similar results were found in OCL-LY-19 ABT199-R and inherently resistant DOHH2 cells (Figures 6e and f and data not shown). Individual treatment with RAD001 and GS-1101 had no effect on parental and a moderate effect on resistant cells at the higher concentrations.

**Figure 7:**
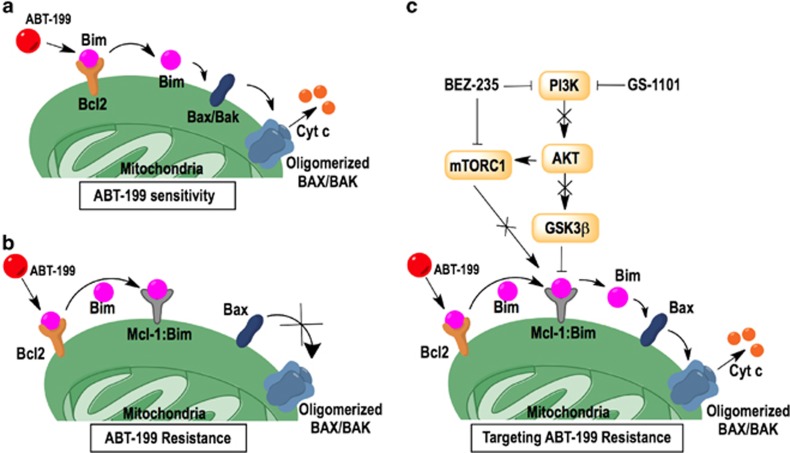
Model for ABT-199-resistance and PI3K-mTOR-mediated targeting of ABT-199-R cells. (**a**) ABT-199 targets BCL-2 in sensitive cells and displaces BIM to cause apoptosis through BAX activation. (**b**) ABT-199 does not target MCL-1 and BCL-xL, which confers resistance by sequestering BIM displaced from BCL-2. (**c**) NVP-BEZ235 inhibits the PI3K and mTOR pathways, which interfere with MCL-1 stability, thereby freeing BIM, which then activates BAX, leading to release of cytochrome *c* to cause cell death

To gain a better understanding of the signaling proteins that are responsible for the GS-1101+ABT-199 sensitization, we treated SU-DHL-6 and OCL-LY-19 ABT199-R cells with ABT-199+GS-1101 for 24 h. Immunoblotting analysis show decreased MCL-1 expression with this combination, with no changes observed in BCL-2 and BCL-xL expression levels. The decrease in MCL-1 corresponded to an increase in cleaved caspase-3 (Figure 6g). AKT activity decreased following GS-1101 addition alone, which was more pronounced with the ABT-199 combination in resistant cells. BAX was activated in ABT199-R cells only with the combination treatment (Figure 6f). Taken together, these findings indicate that the GS-1101+ABT-199 combination exploited the resistant cell's dependency on the PI3K/mTOR pathway by decreasing MCL-1 expression through regulating AKT activity to cause apoptosis (Figure 7).

## Discussion

Rational drug development must anticipate potential mechanisms of resistance, both intrinsic and acquired, in order to develop biomarkers to predict patient response, stratify patients, and to design combination regimens with increased efficacy and applicability. Here, we have addressed the emerging concern that resistance develops to novel BH3 mimetics in lymphoid malignancies and have demonstrated rational combination approaches to overcome it based on the resistant determinants developed. Navitoclax, a small molecule BH3 mimetic, was designed to function as a dual inhibitor against BCL-2 and BCL-xL in refractory CLL.^11, 16, 24^ BCL-xL inhibition in clinical settings led to dose-dependent thrombocytopenia that prompted redesigning of navitoclax into a BCL-2-specific inhibitor, ABT-199. This second-generation inhibitor has a strong affinity for BCL-2, with no effect on human platelets.^17^ Our results show that as a single agent, ABT-199 effectively induced apoptosis in DLBCL cell lines in the nanomolar concentration range as early as 4 h after treatment. Similar results were reported for AML primary cells and mouse models.^19^ However, ABT-199 was less effective against cell lines that had high intrinsic MCL-1 and BCL-xL expression. In our efforts to understand the mechanism of ABT-199-resistance, we developed ABT-199-R variants from ABT-199-sensitive DLBCL cell lines. On comparing expression of BCL-2 family members between ABT199-R and parental cells, we found that the constitutive expression of MCL-1 and BCL-xL was a key feature in maintaining ABT-199-resistance.

MCL-1, an essential cellular protein,^34^ has a short half-life that distinguishes it from BCL-2 and BCL-xL. The half-life of MCL-1 depends on the phosphorylation of residues on its PEST domain that determines its enhanced ability to sequester BIM and also regulates its proteasomal-mediated degradation.^28^ In addition to increased steady state *Mcl-1* and *Bcl-xL* mRNA levels, MCL-1 protein was also more stable in ABT199-R cells. BIM was predominantly associated with BCL-2 in parental cells, which made them sensitive to ABT-199. In contrast, the high MCL-1 and BCL-xL expression in ABT199-R cells sequestered BIM and made them resistant to chronic ABT-199 exposure (Figure 7). A recent study has reported the presence of mutations in BCL-2 and BAX in ABT-199-R mouse and human cell lines, respectively, that conferred ABT-199-resistance.^35^ Although we did not explore the mutational status in our resistance model, the fact that BIM was associated with MCL-1 and BCL-xL, but not BCL-2, indicates that MCL-1 and BCL-xL play a major role in ABT-199-resistance. Moreover, BIM binding to MCL-1 and BCL-xL in ABT199-R cells did not change either in the presence or absence of ABT-199, indicating that ABT-199-resistance was irreversible. It should be noted that with acute ABT-199 treatment MCL-1, but not BCL-xL, level was increased in parental cells. However, this transient increase in MCL-1 level could not capture and sequester BIM that ABT-199 displaced from BCL-2, nor could it prevent cell death by BAX activation and cleavage of caspase-3. These findings indicate the importance of BCL-xL along with MCL-1 in mediating ABT-199-resistance.

B-cells, circulating and those residing in the lymph node niche, receive critical survival, adhesion, and proliferative signals from B-cell receptor signaling through the PI3K/AKT/mTOR pathway. These signals are specifically mediated by the delta isoform (PI3K*δ*) of PI3K that is mainly expressed on B cells.^8, 26, 36, 37^ The PI3K/AKT/mTOR pathways regulate MCL-1 by 4EBP1/mTORC1-dependent translation, AKT/GSK3-*β*-dependent ubiquitination followed by proteasomal-mediated degradation, and AKT/STAT3- or AKT/CREB-mediated transcription.^9, 25, 38, 39, 40^ Hence, we examined whether the apoptotic potential of ABT-199 would be enhanced by combining it with PI3K/AKT/mTOR inhibitors, particularly in cells with elevated MCL-1 and BCL-xL levels. We recently reported that malignant B cells resistant to fludarabine have increased mTOR activity that can be effectively targeted to overcome their resistance.^33^ AKT activation was, indeed, elevated in ABT199-R cells, thus making them more sensitive to simultaneous inhibition of the PI3K/AKT pathway (by the PI3K/AKT/mTOR inhibitor NVP-BEZ235 or the PI3K*δ* inhibitor GS-1101) and BCL-2 (by ABT-199). Similar results were obtained with a combination approach in cells that were inherently resistant to ABT-199 owing to their high MCL-1 and BCL-xL protein expression.

Mechanistically, NVP-BEZ235+ABT199 and GS-1101+ABT199 treatments could downregulate MCL-1, releasing BIM sequestered by MCL-1 and leading to BAX activation (Figure 7). This approach provides a more clinically amenable and effective alternative to using pan-BCL-2 family inhibitors, such as obatoclax or gossypol,^24, 41^ or specific inhibitors on MCL-1, such as maritoclax^42^ or UMI-77.^43^ *In vivo* experiments with GS-1101 have shown that it can block the B-cell receptor signaling pathway by inhibiting PI3K and preventing activation of its downstream targets AKT and ERK.^36, 44^ In addition to the potential intracellular benefits of this combination, clinically, the GS-1101+ABT-199 combination could have another benefit. In the absence of critical signals via the B-cell receptor, CLL cells mobilize into the blood stream, where they may be more ‘primed for death'^36^ and hence would be sensitive to ABT-199. A similar effect can be expected for NVP-BEZ235, which inhibits both PI3K and mTOR (mTORC1 and mTORC2) pathways. In fact, this dual inhibition also targets feedback signaling that regulates AKT activation. Indeed, cell death in response to NVP-BEZ235+ABT199 was more robust with inhibition of AKT and mTORC1 (as seen by evaluating its downstream targets p-4EBP1 and p-p70S6 kinase) and corresponding downregulation of MCL-1. Interestingly, the combination treatment did not lower BCL-xL expression in ABT199-R cells, which would be beneficial in the clinic, as it would probably not lead to thrombocytopenia in patients as reported with navitoclax.^16^ It should be noted that although both BCL-xL and MCL-1 were required for developing acquired ABT-199-R, targeting only one sensitized resistant cells to ABT-199. Suppression of MCL-1 expression with PI3K/AKT/mTOR inhibitors was not, however, sufficient to tip the balance toward cell death unless ABT-199 was added.

In summary, combining PI3K/AKT/mTOR inhibitors with ABT-199 disturbed the critical equilibrium between anti-apoptotic and BH3-only proteins, lowering the threshold needed to activate apoptosis. Our findings provide new insights into the molecular mechanisms of ABT-199-resistance and indicate the potential for combining ABT-199 with PI3K/AKT/mTORC inhibitors to lower ABT-199 dose and enhance its applicability in solid tumors as well.

## Materials and Methods

### Cell lines and reagents

Human leukemic cell lines SU-DHL-6, OCL-LY-19, SU-DHL-16 (DLBCL), and DOHH2 (FL) were obtained from Dr. Eric Hsi and Dr. Neetu Gupta (Cleveland Clinic), Nalm6, Reh (acute lymphoblastic leukemia) were purchased from the American Type Culture Collection (Manassas, VA, USA), Mec-2 from Dr. Y. Saunthararajah (Cleveland Clinic) and MO1040 (both CLL) from Dr. Riccardo Dalla-Favera (Columbia University) and used as before.^32, 33, 45^ All cell lines were cultured in RPMI-1640 medium supplemented with 10% FBS (Atlanta Biologicals, Lawrenceville, GA, USA), and antibiotic-antimycotic (Gibco, Life Technologies, Gaithersburg, MD, USA). ABT199-R cells were cultured with 5% FBS. Cell lines were routinely screened for *Mycoplasma*, variations in growth rates, changes in morphological characteristics, and their response to stress with Annexin V FITC-PI staining; their passage number did not exceed 20. ABT-737 and ABT-199 were obtained from AbbVie (Chicago, IL, USA); NVP-BEZ235, RAD001, and GS-1101 from Selleck Chemicals (Houston, TX, USA); and verapamil and cycloheximide from Sigma-Aldrich (St. Louis, MO, USA).

### Generation of ABT-199-R cell lines

The DLBCL cell lines, SU-DHL-6 and OCL-LY-19, were made resistant to ABT-199 as previously described.^24^ Briefly, cells were intermittently incubated with a low concentration (fivefold lower than IC_50_) of ABT-199 for short intervals over time and allowed to recover after washing off the drug. The ABT-199 concentration and treatment time were gradually increased until cells remained viable after a continuous exposure to the drug that was double the concentration of their IC_50_ value. To make sure that cells were not becoming resistant via increased expression of efflux pumps, cells were treated intermittently with verapamil. The ABT199-R cells were routinely tested for resistance to ABT-199 and cultured without drug for 72 h before they were used in experiments.

### Flow cytometry

Cell viability was measured by phosphatidylserine externalization by staining the cells with fluorescein-conjugated Annexin V-PI (BD Biosciences, San Jose, CA, USA). The analysis was done on a BD FACS Calibur flow cytometer (BD Biosciences), and the raw data was processed using CellQuest Version 5.2.1 software (BD Biosciences, Franklin Lakes, NJ, USA). The results were normalized to survival of vehicle control cells treated with ethanol or dimethyl sulfoxide.

### Immunoblotting and immunoprecipitation

The cell pellets were lysed with 1% NP-40 lysis buffer 20 mmol/l Tris-HCl, pH 7.5; 1 mmol/l EDTA; 150 mmol/l NaCl; 1% NP-40), containing phosphatase inhibitor cocktails 2 and 3 (Sigma) and protease inhibitors (Roche, Indianapolis, IN, USA), for 30–45 min at 4 °C. Protein lysates were prepared after calculating protein concentration using Bradford reagent (Bio-Rad, Hercules, CA, USA); 50 *μ*g of protein was resolved on 10–12% SDS-PAGE followed by transferring to nitrocellulose or PVDF membranes (Millipore, Danvers, MA, USA).^24^ The immunoblotting was performed with the primary antibodies mentioned below. For immunoprecipitation, protein lysates were prepared by lysing cell pellets with CHAPS buffer (20 mmol/l Tris-HCl, pH 7.5; 150 mmol/l NaCl; 1 mmol/l EDTA; 2% CHAPS; Calbiochem, Billerica, MA, USA) containing protease and phosphatase inhibitors for 1 h on ice. Protein lysates were incubated with primary antibody overnight at 4 °C, then an equal amount of protein A agarose beads (Calbiochem) was added to all samples, followed by 1 h of incubation at 4 °C. The beads were washed three times with CHAPS, eluted with loading buffer supplemented with 2-mercaptoethanol (Sigma-Aldrich) and western blotting was performed as mentioned above. BAX activation was determined as described elsewhere.^24^ Primary antibodies used were for MCL-1, BIM, BAX-6A7 (BD Biosciences), NOXA (Enzo Life Sciences, Farmingdale, NY, USA), BCL-2, BCL-xL, BAX-N20 (Santa Cruz Biotechnology, Santa Cruz, CA, USA), PUMA (ProSci Incorporated, Poway, CA, USA), cleaved caspase-3, total-AKT, p-AKT (Ser473), p-AKT (Ser308), total-p-70S6 Kinase, p-p70S6 Kinase (Thr389), p-4EBP1 (Ser65) (Cell Signaling Technologies, Danvers, MA, USA), and *β*-actin (Sigma). The secondary anti-mouse and -rabbit antibodies were purchased from Thermo-Fisher Scientific (Pittsburg, PA, USA). Protein levels were quantified by ImageJ (NIH, Bethesda, MD, USA); the relative intensity of each lane with respect to control at 0 h was calculated after normalizing it to the relative intensity of *β*-actin.

### RNA isolation and real-time quantitative-PCR

RNA was isolated by the Trizol method (Life Technologies) from parental and ABT199-R cells after ABT-737 treatment. Levels of mRNA were analyzed using a quantitative real-time, reverse transcriptase PCR (qRT-PCR) kit (Life Technologies), with primers for *Mcl-1*, *Bcl-xL*, and *Bcl-2*, and normalized for β*-actin*, as described.^15, 24^

### siRNA transfection

AKT, MCL-1, and BCL-xL knockdown was achieved using specific siRNA or siControl (Santa Cruz Biotechnology) by Amaxa Nucleofector Kit V (Lonza, Walkersville, MD, USA) (program number O-007) according to the manufacturer's protocol.

### Statistical analysis

Statistical comparisons between groups were conducted by student *t*-test and protein half-life was analyzed using one-phase exponential decay model in Prism (Version 4.0c, GraphPad software Inc, La Jolla, CA, USA). The standard deviation was calculated from experiments conducted in triplicate and is indicated by error bars on the figures. All experiments were repeated three times independently.

## Abbreviations

ABT199-R: ABT-199 resistant
BAX: Bcl-2-associated X protein
BAK: Bcl-2 homologous antagonist/killer
BIM: BCL-2-interacting mediator of cell death
BCL-2: B-cell lymphoma 2
BCL-w: BCL-2-like protein w
Bcl-xL: B-cell lymphoma-extra large
BH3: BCL-2 homology-3
CLL: chronic lymphocytic leukemia
MCL-1: myeloid cell leukemia 1
mTOR: mechanistic target of rapamycin
NOXA: phorbol-12-myristate-13-acetate-induced protein 1
PI: propidium iodide
PI3K: phosphatidylinositol 3-kinase
PUMA: p53 upregulated modulator of apoptosis
qRT-PCR: quantitative real-time polymerase chain reaction
siRNA: small interfering RNA
