# Correction to: MCL-1 and BCL-xL-dependent resistance to the BCL-2 inhibitor ABT-199 can be overcome by preventing PI3K/AKT/mTOR activation in lymphoid malignancies

**DOI:** 10.1038/s41419-024-06878-1

**Published:** 2024-07-24

**Authors:** G. S. Choudhary, S. Al-harbi, S. Mazumder, B. T. Hill, M. R. Smith, J. Bodo, E. D. Hsi, A. Almasan

**Affiliations:** 1 Department of Cancer Biology, Lerner Research Institute, Cleveland Clinic, Cleveland, OH 44195 USA; 2 Department of Pathology, Case Western Reserve University School of Medicine, Cleveland, OH 44106 USA; 3 Department of Hematology and Oncology, Taussig Cancer Institute, Cleveland Clinic, Cleveland, OH 44195 USA; 4 Department of Clinical Pathology, Institute of Pathology and Laboratory Medicine, Cleveland Clinic, Cleveland, OH 44195 USA

Correction to: *Cell Death & Disease* 10.1038/cddis.2014.525, published online 15 January 2015

In this article Figs. 5 and 6 have been corrected:

Figure 5 correction: The loading controls for Akt and T-p70S6 kinase in Fig. 5A were inadvertently duplicated. We have located the blots for total -Akt and total p70S6 kinase in SUDJL-6-ABT199 cells (S199R) and corrected Figure 5. Total Akt and p70S6 kinase levels were not changed when treated with ABT199, BEZ235, or in combination. p70S6 kinase and 4EBP1 are downstream targets of Akt/mTORC1.
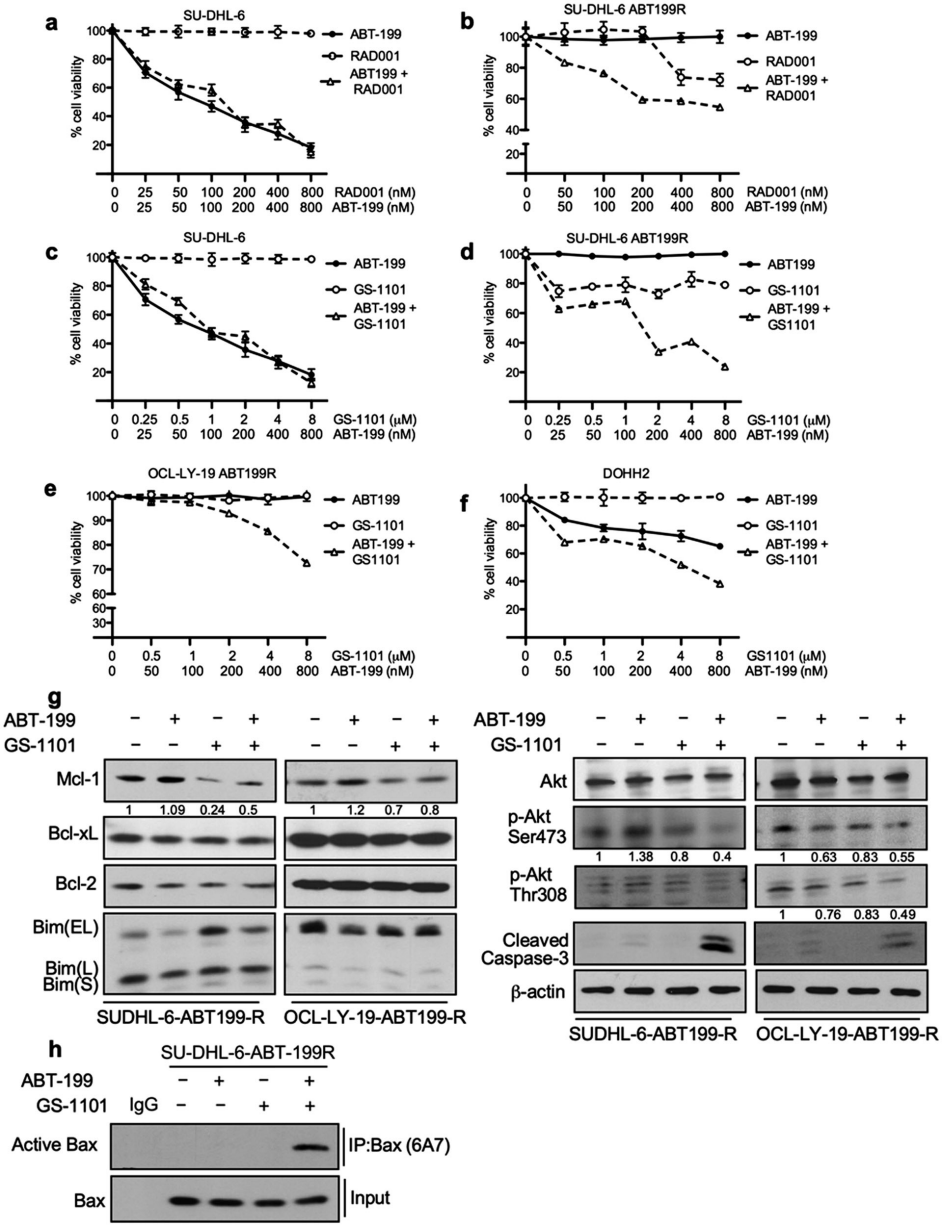


The original article has been corrected.
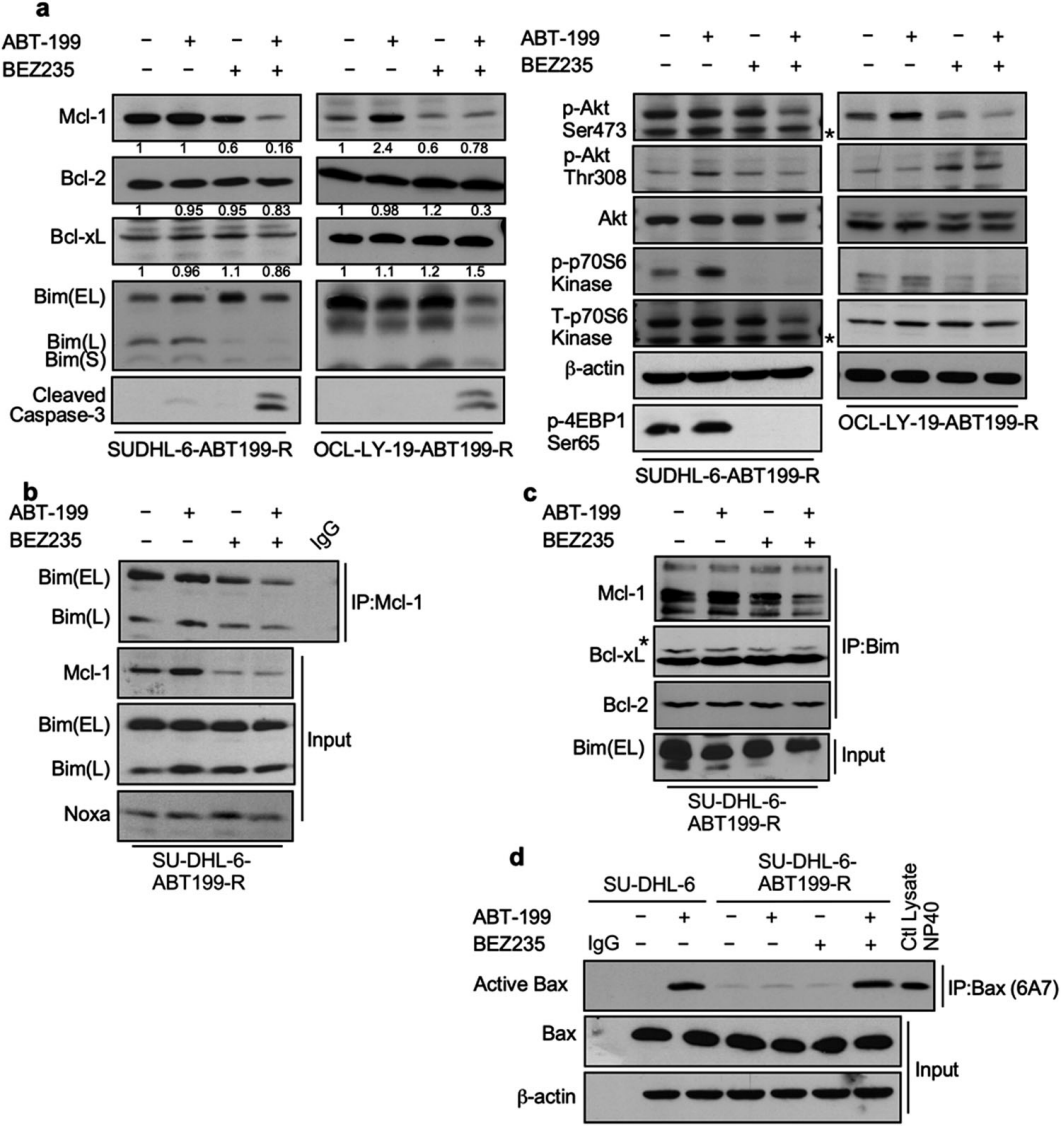


Figure 6 correction: Akt is phosphorylated more in ABT-199 resistant cells as compared to parental control cells as shown in Fig. 1e. When SUDHL6-ABT-R cells are treated with ABT-199, p-Akt levels are increased. This phosphorylation is confirmed independently either by p-AktSer473 or p-AktThr308 signals. We have inadvertently placed the same blot of p-AktThr308 in Figs. 5a and 6g. We have located the blots for p-Akt (ABT-199+BEZ235) and p-Akt (ABT-199+GS-1101) in SUDJL-6-ABT199 cells (S199R) and corrected Figure 6.

