# Supplementary figures and images for: MCL-1 and BCL-xL-dependent resistance to the BCL-2 inhibitor ABT-199 can be overcome by preventing PI3K/AKT/mTOR activation in lymphoid malignancies
Source: Cell Death Dis. 2015 Jan 15;6(1):e1593–. doi: 10.1038/cddis.2014.525 (PMC4669737; doi:10.1038/cddis.2014.525)

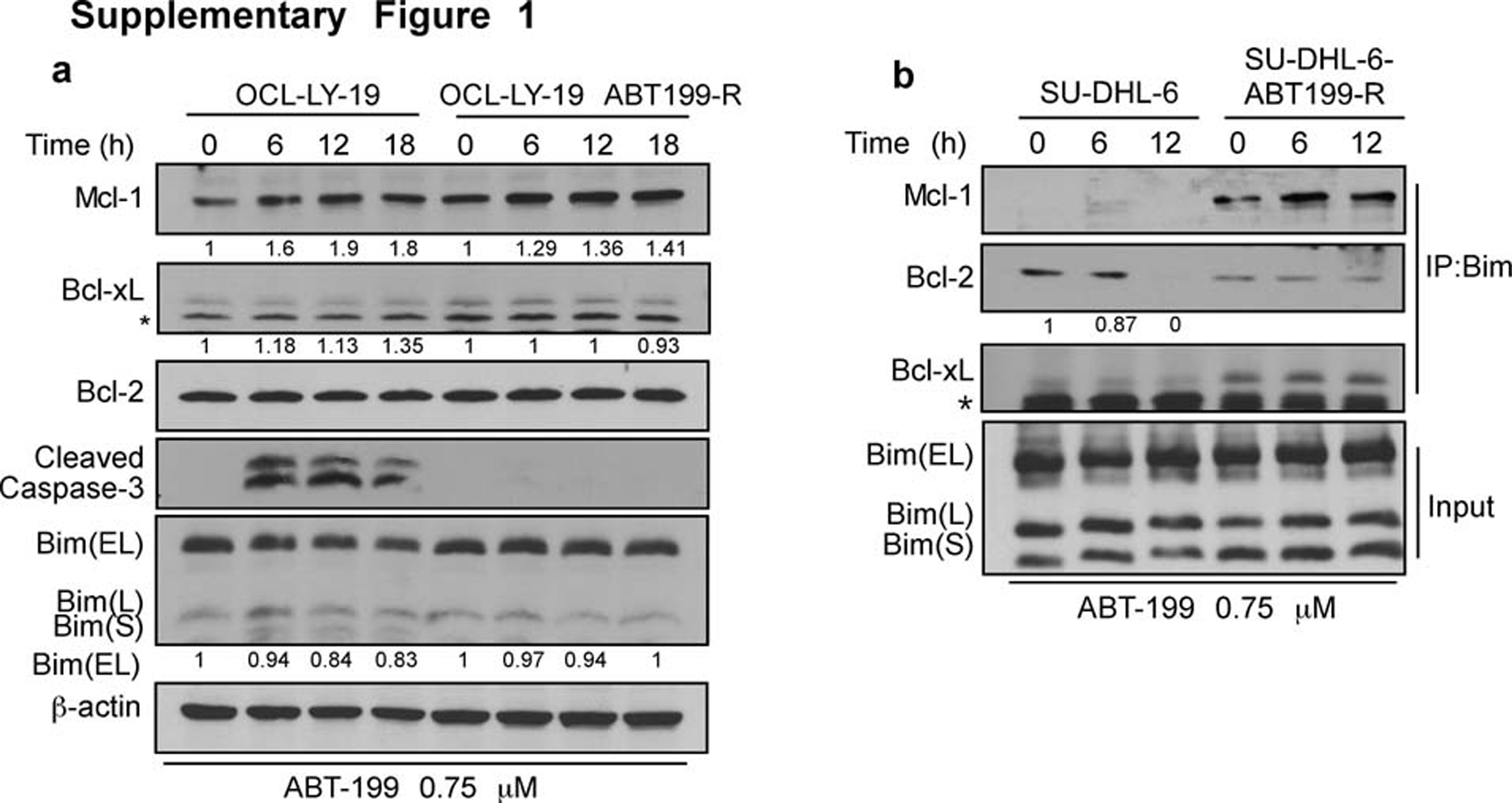

Supplement: Supplementary Figure 1 [file cddis2014525x1.tif]

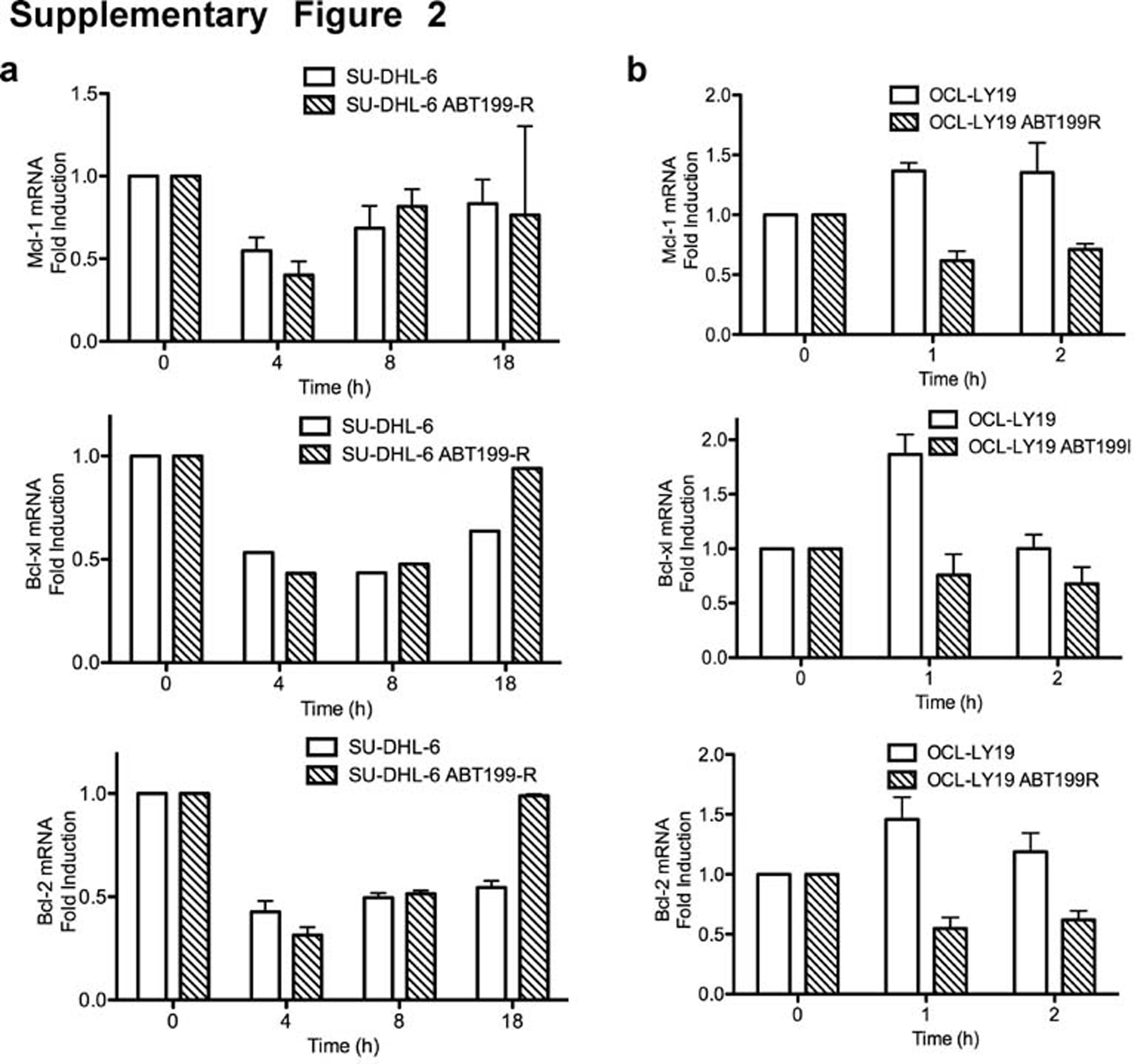

Supplement: Supplementary Figure 2 [file cddis2014525x2.tif]
