# Supplementary material for: MCL-1 and BCL-xL-dependent resistance to the BCL-2 inhibitor ABT-199 can be overcome by preventing PI3K/AKT/mTOR activation in lymphoid malignancies
Source: Cell Death Dis. 2015 Jan 15;6(1):e1593–. doi: 10.1038/cddis.2014.525 (PMC4669737; doi:10.1038/cddis.2014.525)
Supplement: Supplementary Figure Legends [file cddis2014525x3.doc]

**Supplementary Figures**

**Supplementary Figure S1**

**Elevated MCL-1 and BCL-xL levels in ABT199-R cells sequester Bim. (a)** OCL-LY-19 parental and ABT-199R cells were treated with ABT-199 for the indicated time. MCL-1, BCL-2, BCL-xL, BIM, and cleaved caspase-3 levels were determined by immunoblotting. β-actin was used as a loading control. **(b)** SU-DHL-6 parental and resistant cells were treated with ABT-199 at the indicated time, and BIM immunoprecipitates were examined by immunoblotting for association with MCL-1, BCL-2, and BCL-xL

**Supplementary Figure S2**

**No consistent changes in *Mcl-1*, *Bcl-xL*, and *Bcl-2* mRNA levels in response to acute ABT-199 treatment.** RNA was extracted at indicated time points from parental and ABT199-R **(a)** SU-DHL-6 and **(b)** OCL-LY-19 cells treated with ABT-199, and the fold change for *Mcl-1*, *Bcl-xL*, and *Bcl-2* was determined by quantitative real-time PCR. RNA levels of control cells treated with DMSO were set to 1.
